# Case report: Pathological and genetic features of pancreatic undifferentiated carcinoma with osteoclast-like giant cells

**DOI:** 10.3389/pore.2023.1610983

**Published:** 2023-03-03

**Authors:** Ni Zhao, Nan Mei, Ye Yi, Hongyan Wang, Yajian Wang, Yu Yao, Chunli Li

**Affiliations:** ^1^ Department of Medical Oncology, The First Affiliated Hospital of Xi’an Jiaotong University, Xi’an, Shaanxi Province, China; ^2^ Department of Hematology, The First Affiliated Hospital of Xi’an Jiaotong University, Xi’an, Shaanxi Province, China; ^3^ Department of Pathology, The First Affiliated Hospital of Xi’an Jiaotong University, Xi’an, Shaanxi Province, China

**Keywords:** prognosis, pathological features, undifferentiated carcinoma, osteoclast-like giant cells, pancreatic carcinoma

## Abstract

**Objectives:** Pancreatic undifferentiated carcinoma accounts for 2%–7% of pancreatic carcinomas. We aimed to investigate the pathological and genetic characteristics of pancreatic undifferentiated carcinoma with osteoclast-like giant cells and the key points of treatment.

**Methods:** The clinical data and follow-up results of four patients diagnosed with pancreatic undifferentiated carcinoma with osteoclast-like giant cells between May 2015 and May 2020 at the First Affiliated Hospital of Xi’an Jiaotong University were retrospectively analyzed.

**Results:** Chief complaints included “pain and discomfort in the upper abdomen” (2/4), “nausea and vomiting” (1/4) or no symptoms (1/4). Preoperative mildly elevated tumor markers included carcinoembryonic antigen (1/4) and CA19-9 (1/4). The tumors were located in the tail of the pancreas in three patients and the head and neck in one patient. Tumor metastasis was found in pancreatic adipose tissue in two patients and lymph node metastasis in one patient, with microscopic heterogeneous mononuclear cells and scattered osteoclast-like giant cells of various sizes. One patient (1/4) had a mucinous cystic tumor of the pancreas, and two patients (2/4) had adenocarcinoma of the pancreatic duct. Only one patient received postoperative gemcitabine combined with albumin-bound paclitaxel chemotherapy.

**Conclusion:** Currently, treatment guidelines are lacking for PUC-OGC, and prognosis varies markedly. More cases must be reported to clarify its origination. The long-term follow-up of diagnosed patients and genetic mutation testing can also contribute to improving treatment and prognosis of this disease.

## Introduction

Pancreatic undifferentiated carcinoma with osteoclast-like giant cells (PUC-OGC), characterized by monocytes and osteoblast-like giant cells in the tumor tissue and first reported by Rosai in 1968 (1), is even rarer, with an incidence of less than 1% (2) of all pancreatic malignancies. There is a great controversy about its histological origin and diagnosis is exclusively based on histopathological examination of biopsies. Due to the lack of specific clinical manifestations or preoperative diagnostic tests, early diagnosis and treatment modalities are still challenging. Many cases reach advanced stages at the time of detection. Currently, there are no treatment guidelines, and the prognosis of different interventions varies markedly. Long-term follow-up of diagnosed and surviving patients and genetic testing of mutated genetic loci may contribute to guide the selection of treatment options and prognostic analysis. Modern genetic sequencing techniques such as Next-Generation Sequencing (NGS) have become widespread to better meet the needs of patients with cancer, particularly those with rare or advanced diseases. More than two-thirds of medical oncologists in the United States report having used NGS to inform clinical care decisions (3). In this study, we collected clinical data from four patients with PUC-OGC admitted to the First Affiliated Hospital of Xi’an Jiaotong University and outlined their pathological and genetic characteristics in the context of medical literature.

## Data and methods

Clinical data of four patients diagnosed with PUC-OGC in the First Affiliated Hospital of Xi’an Jiaotong University from May 2015 to May 2020 were collected and retrospective analysed, including the patients’ general conditions (age, gender, symptoms, presence of weight loss, history of diabetes mellitus, history of smoking and drinking), results of adjuvant examinations (abdominal ultrasonography, three-stage enhanced Computed Tomography(CT) of the pancreas, preoperative and postoperative tumor markers), histological characteristics (tumor texture, tumor location, pathological findings, immunohistochemistry), treatment (implementation of surgical procedure, postoperative adjuvant therapy) and follow-up information.

The study had been performed according to the Declaration of Helsinki and was approved by the Ethics Committee of the First Affiliated Hospital of Xi’an Jiaotong University.

## Results

### Case 1

**FIGURE 1:**
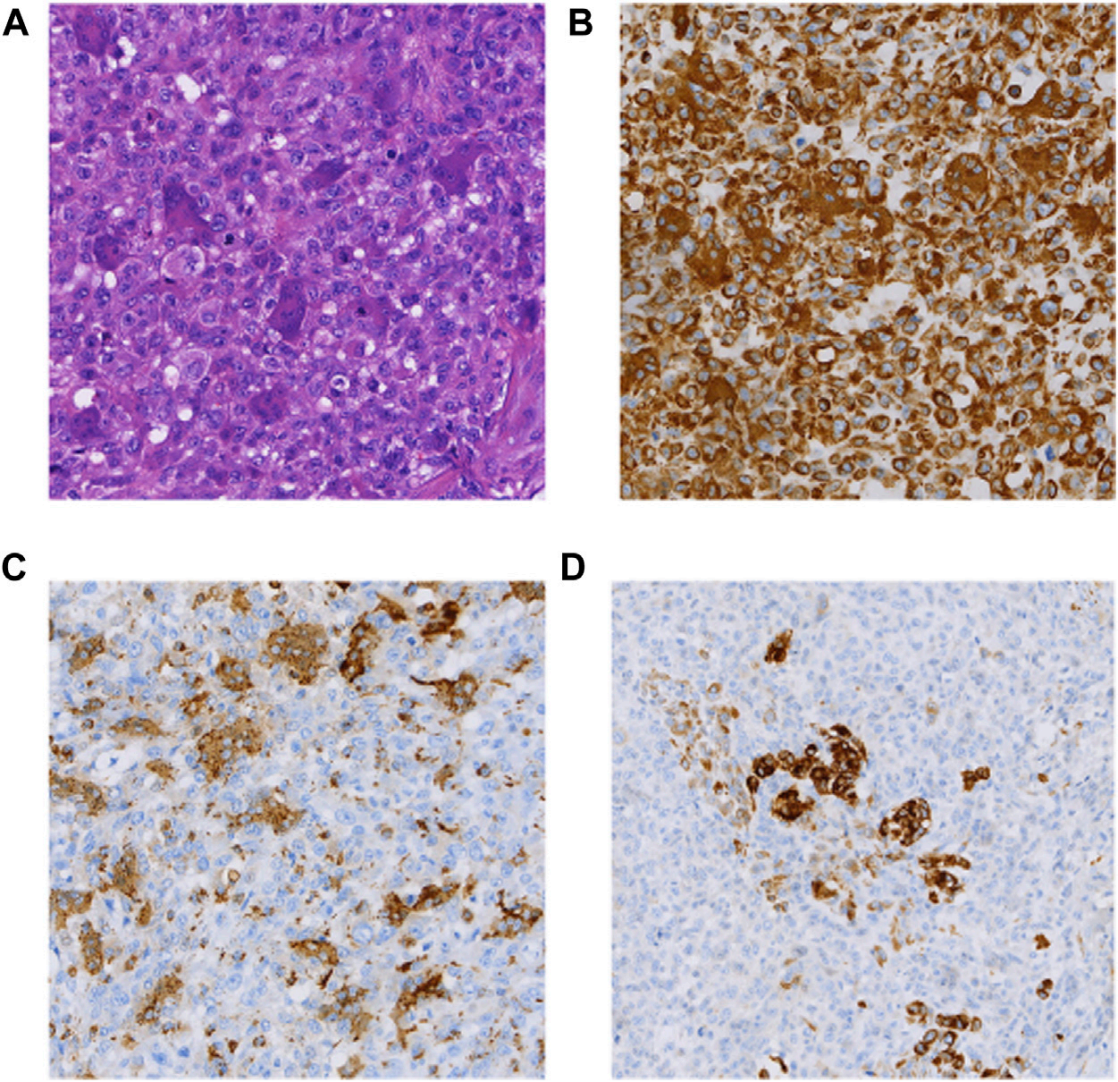
**(A)** HE (×100); **(B)** Vimentin(+) (×100); **(C)** CD68(+) (×100); **(D)** CK(partial+) (×100).

The patient was a 71-year-old man hospitalized due to nausea and vomiting with progressive jaundice within 40 days. He underwent laparoscopic pancreatic body and tail excision with splenic modular resection and partial jejunostomy anastomosis at a local hospital. Macroscopically, an intraoperative occupying lesion at the duodenal papilla was seen. On 24 May 2017, he underwent whipple & splenectomy & radiofrequency ablation of liver metastases as palliative surgery in our hospital. A 3 cm oval-shaped hard mass of pancreatic origin was palpable in the lesser curvature of the stomach. Histomorphologically, the tumor exhibited PUC-OGC (focal ductal adenocarcinoma component). Immunohistochemical staining demonstrated the following (Figure 1): CK (cytokeratin) (partial +), CK20 (−), CK19 (partial +), vimentin (Vim) (+), alpha-smooth muscle actin (SMA) (focal +), desmin (DES) (−), CD34 (−), P63 (partial +), CD68 (osteoblast-like giant cell +), P53 (+5%), Ki67 (+80%), and epithelial membrane antigen (EMA) (partial +). The patient died 10 months postoperatively without receiving any adjuvant treatment.

### Case 2

**FIGURE 2:**
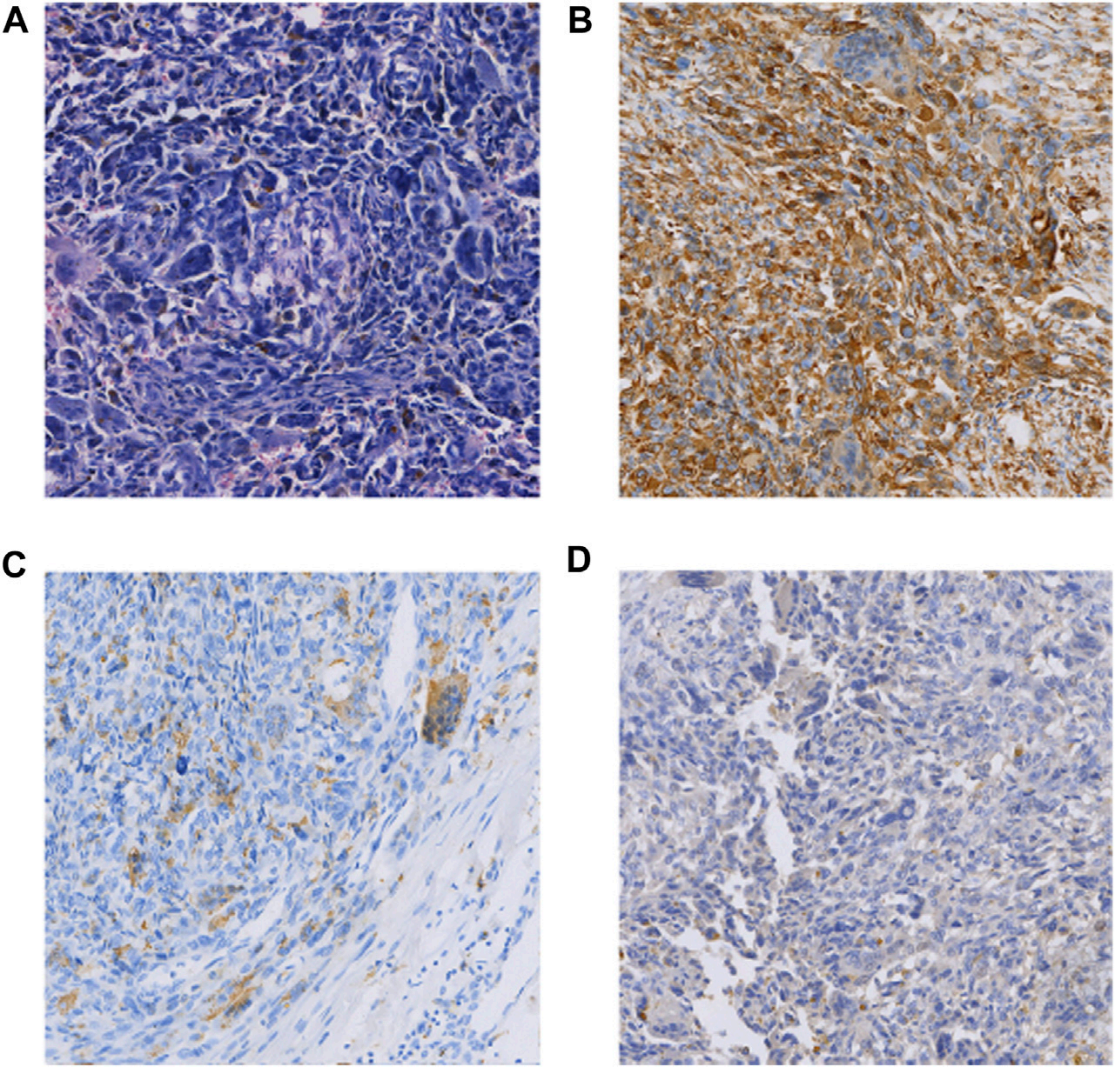
**(A)** HE (×100); **(B)** Vimentin(+) (×100); **(C)** CD68(focal +) (×100); **(D)** CK(focal +) (×100).

The patient was a 65-year-old man who underwent pancreaticoduodenectomy and cholecystectomy in our hospital on 13 September 2017. A hard mass with a diameter of approximately 5.0 cm in the posterior hook of the pancreatic head, descending part of the duodenum and horizontal part of the pancreas, which was clearly demarcated from the surrounding tissues. Histomorphologically, the mass was pancreatic polymorphic malignant tumor tissue. The structure of the slice combined with the immunohistochemical results suggested PUC-OGC invading the entire intestinal wall of the duodenal papilla. Immunohistochemical staining showed the following (Figure 2): cytokeratin (CK) (focal +), EMA (focal +), Vim (+), CD117 (−), CD34 (−), SMA (focal +), S100 (small focal +) CD68 (focal +), Bcl-2 (+), CA199 (−), and Ki67 (+30%). The patient was followed up until October 2020 and did not receive any postoperative adjuvant therapy, and regular review showed no new lesions or distant metastases.

### Case 3

**FIGURE 3:**
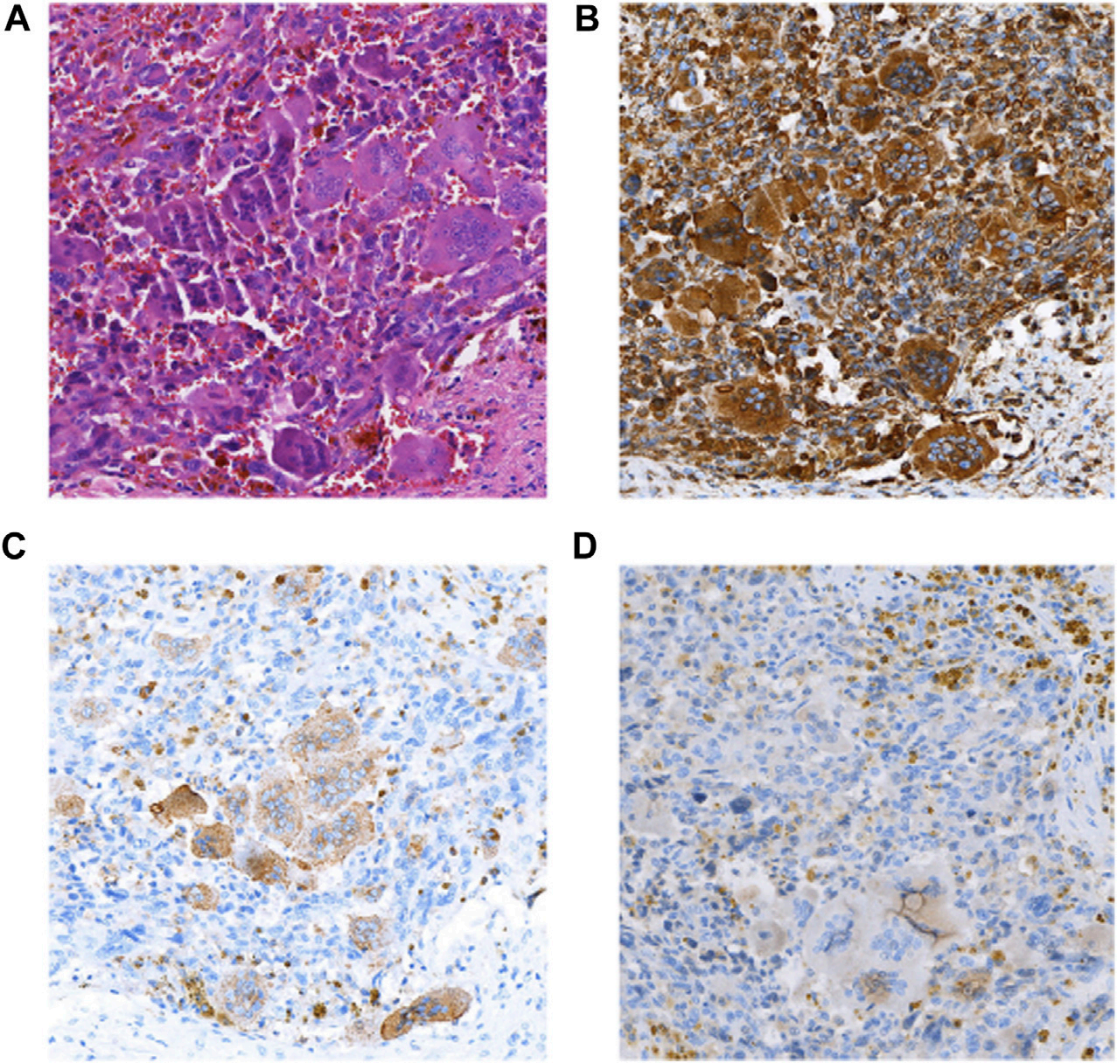
**(A)** HE(×100); **(B)** Vimentin(+) (×100); **(C)** CD68(OGC+) (×100); **(D)** CK(+) (×100).

The patient was a 46-year-old man with intermittent left upper abdominal distension and pain with intermittent diarrhea who underwent abdominal CT at a local hospital showing pancreatic occupancy. On 1 April 2020, he underwent pancreatic tail resection and splenectomy in our hospital. Intraoperatively, a hard mass of approximately 4 cm in diameter could be planted in the neck of the pancreas, which was closely adherent to the portal vein. Histomorphologically, the tumor mixed undifferentiated carcinoma of the pancreas with osteoblast-like giant cells and a medium-differentiated ductal adenocarcinoma component, which accounted for approximately 30% of the mass. The tumors invaded peripancreatic fibrofatty tissue and parasplenic tissue, and no metastasis was seen in the lymph nodes sent for examination. Immunohistochemical staining showed the following that were (Figure 3) CK (adenocarcinoma component+), CK7 (adenocarcinoma component+), Vim (+), CD68 (osteoblast-like giant cells+), P63 (focal+), and Ki67 (+30%). The patient died 1 month after surgery receiving any postoperative adjuvant therapy.

### Case 4

**FIGURE 4:**
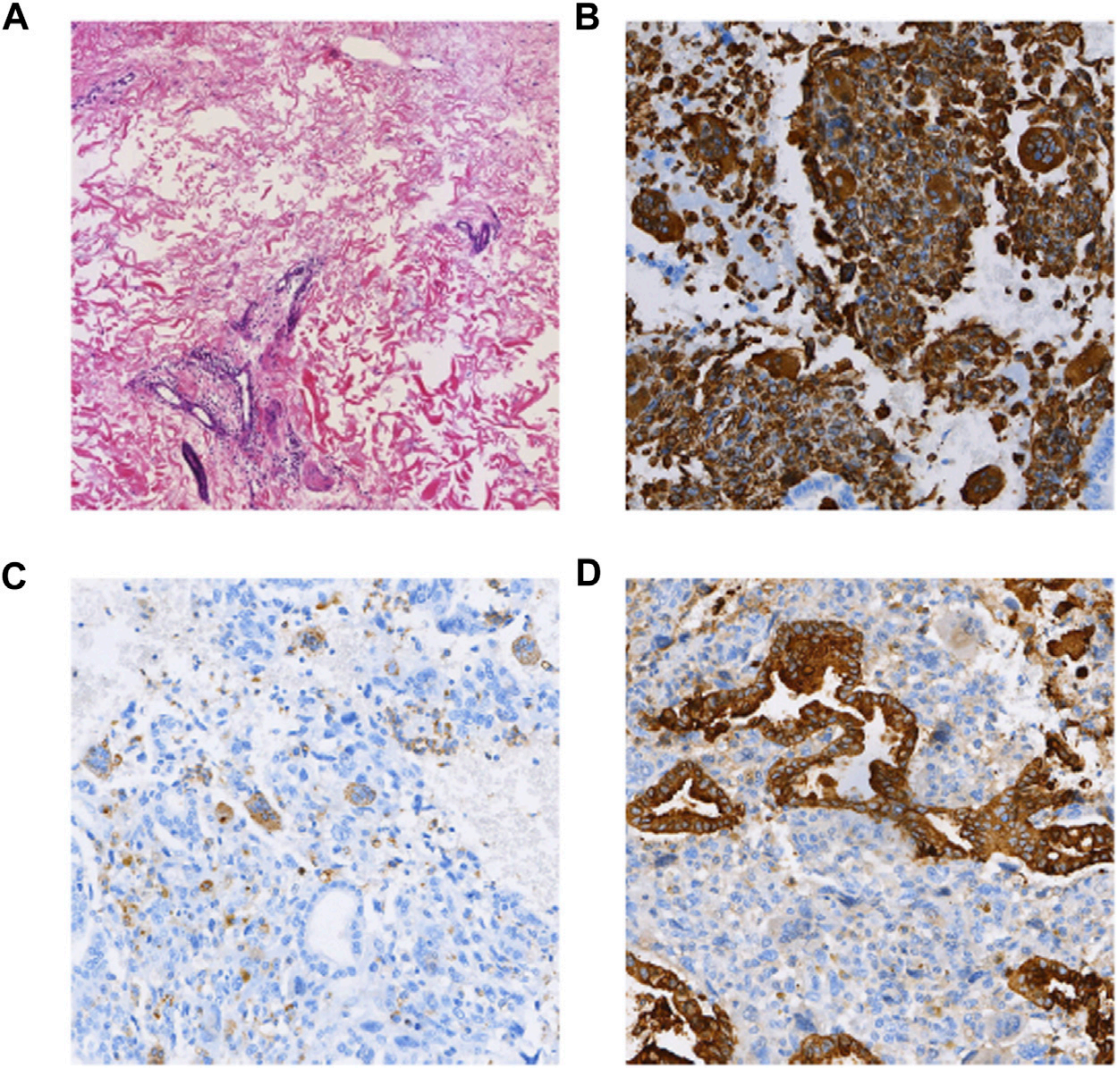
**(A)** HE (×100); **(B)** Vimentin (intraepithelial carcinoma -, anaplastic +) (×100); **(C)** CD68(+) (anaplastic +) (×100); **(D)** CK(intraepithelial carcinoma +, few anaplastic +) (×100).

A 37-year-old woman was admitted to the hospital with recurrent epigastric distension for 2 months. On 15 February 2020, an abdominal ultrasound was performed at a local hospital, and the result suggested a cystic mass in the tail of the pancreatic body. Abdominal CT showed that the morphology of the pancreas was altered with caudal occupancy of the pancreas, the descending colon was compressed with possible adhesions, and the splenic artery and vein were altered after compression. One week later, she underwent resection of pancreatic body, tail and splenectomy at a local hospital, during which a mass of approximately 12 cm × 5 cm × 5 cm was observed in the body caudal part of the pancreas. Histomorphologically, the tumor exhibited a pleomorphic cell component, namely an admixed mucinous cystic tumor of the pancreas with papillary intraepithelial carcinoma with undifferentiated pancreatic cancer with osteoblast giant cells. The tumor invaded the peripancreatic adipose tissue; however, tumor involvement in the spleen and lymph nodes was not obseved. Immunohistochemical staining (as in Figure 4) revealed the following Vim (intraepithelial carcinoma -, anaplastic +), CK (intraepithelial carcinoma +, few anaplastic +), EMA (intraepithelial carcinoma +), CK7 (intraepithelial carcinoma +), CK8/8 (intraepithelial carcinoma +), CD68 (anaplastic +), CK20 (−), CR (subepithelial mesenchymal +), des (subepithelial mesenchymal +), Muc-1 (intraepithelial carcinoma +), Muc-2 (−), P53 (anaplastic +20%), and Ki-67 (intraepithelial carcinoma +80%, anaplastic +70%). The patient was administered postoperative gemcitabine & albumin-bound paclitaxel combination chemotherapy for 6 cycles, followed by regular follow-up until December 2020, and no recurrence or metastasis was detected.

Pathological sections from this patient were subjected to whole genome anslysis by second-generation sequencing (Sidi Precision Medicine, Illumina Nextseq500; 3DMed, Shanghai, China, http://www.3dmedcare.com), and the results demonstrated (Supplementary Table S1; Supplementary Figure S1) that 6 genetic mutations were associated with targeted therapy, prognosis and drug resistance. Among them, 6 somatic variants were of possible clinical significance (Supplementary Table S2): increased androgen receptor (AR) copy number, increased cyclin E1 gene (CCNE1) copy number, increased Bruton’s tyrosine kinase gene (BTK) copy number, and FBXW7p. R465H Exon 9, KRASp. G12R Exon 2, TP53 p. P75Lfs*48 Exon 4. PARP inhibitor-related genes had no mutations (BRCA1/2 no mutations, HRD score 0 (Supplementary Figure S2)). Immunotherapy-related gene mutations were observed negative PD-1 immunohistochemistry results (TPS <1%, CPS = 5) and a low tumor mutational burden (TMB) (Supplementary Figure S3A) at 3.35 Muts/Mb, which occur in less 76% of pancreatic cancer patients. Microsatellite analysis showed microsatellite stability (MSS) (Supplementary Figure S3B) and negative EBV test results. HLA typing was heterozygous. Genetic mutations positively associated with immunotherapy were TP53 and KRAS pathogenic mutations. No genetic mutations detected were negatively associated with immunotherapy.

## Discussion

### Clinical manifestation of the PUC-OGC is not specific

PUC-OGC often occurs in elderly patients with no sex differences. Previous reports have indicated that risk factors for PUG-OGC are different from those for pancreatic ductal adenocarcinoma, with little relevance to smoking or alcohol consumption. PUC-OGC most commonly originates from the body or tail of the pancreas. Pathological features of PUG-OGC commonly include massive hemorrhage and necrosis, occasionally with visible calcification with clear margins. The clinical manifestations of PUC-OGC are non-specific and include abdominal pain, a palpable mass, weight loss, etc. The initial symptoms of the patients in this study were abdominal distension, abdominal pain, nausea, vomiting, progressive jaundice and other discomfort (Table 1). One patient was asymptomatic, while the diagnosis was based on a pancreatic mass found on abdominal MRI. Further endoscopic guided puncture biopsy identified a tumor of mesenchymal tissue origin. After tumor resection, the diagnosis of PUC-OGC was only made by pathological analysis.

**TABLE 1:** Basic situation.

Number		1	2	3	4
Age		71	65	46	37
Sex		Male	Male	male	Female
Symptom		Yellow staining of the skin and sclera	None	abdominal distension	abdominal distension
Change in weight		Decrease 9 kg	Decrease 4 kg	None	None
Time of diabetes		Ten years	One month	None	Two years
Smoke or drink		None	None	Drink^a^	None
Preoperative chemotherapy		None	None	None	None^b^
Postoperative survival^c^		Ten months	Three years	One month	Eight months
CEA (ng/mL)	Preoperative	5.87	1.73	2.51	0.04
	Postoperative	-	-	-	2.03
CA-199 (U/mL)	Preoperative	25.28	10.95	14.88	191
	Postoperative	9.28	-	-	9.21
Size^d^		10 cm × 4.5 cm × 1.5 cm	5 cm × 5 cm	4.5 cm × 3 cm × 2.5 cm	9 cm × 5 cm×5 cm
Location		Body and tail	Head and neck	Body and tail	Body and tail
Tumor texture		Sections are grayish yellow, with parts grayish brown	Dark red, pale yellow alternate, brittle, necrotic hemorrhage	Cystic solid mass, soft, with dark red bloody fluid	This is a cystic mass with a slightly hard, compartmentalized inner wall. The cut surface is grayish yellow and reddish
Histomorphologically		PUC-OGC & PADC	PUC-OGC	PUC-OGC & PADC	PUC-OGC & ductal mucinous adenocarcinoma)
Lymphatic metastasis		Mesenteric lymph node (1/3)	Peripancreatic lymph node (1/2)	Peripancreatic lymph node (11), Hilar lymph node(1)	Peripancreatic lymph node (10)
Distant metastasis		Hepatic metastases	None	None	None

^a^ The patient had a 20-year history of smoking, 30 cigarettes per day, and no history of drinking alcohol.

^b^ Gemcitabine combined with albumin binding paclitaxel regimen.

^c^ Follow up to October 2020.

^d^ All were intraoperative visible masses.

Tips: “-”the patient did not detect this indicator before surgery; CEA, blood carcinoma embryo antigen; AFP, blood alpha-fetoprotein; CA19-9, glycemic antigen 19-9; CA-125, glycemic-like antigen 125.

### The origin of PUC-OGC may be the pancreatic ductal epithelium

The histological origin of PUC-OGC has been controversial. Mesenchymal cells, epithelial cells, acinar cell progenitor cells, ductal cells, undifferentiated progenitor cells, stem cells, and even mucinous cystic neoplasm origins have been considered. However, most cases of PUC-OGC are currently believed to originate from pancreatic ductal epithelium changes in the interepithelial tissue. It has also been reported that PUC-OGC coexists with ductal adenocarcinoma. The latest WHO tumor classification standards regard PUC-OGC as a special subtype of pancreatic ductal adenocarcinoma. PUC-OGC is usually negative for cytokeratin and p53 but positive for vimentin, leukocyte common antigen, and macrophage markers such as KP1. Immunohistochemistry showed the positive expression of Vim, CD68 and CK in the 4 cases investigated in this report. Due to some pathologists’ subjective selection of immunohistochemically labeled antibodies, the source of OGC still needs to be further explored. Establishing the origin of PUC-OGC would be of great significance in guiding the choice of treatment.

### PUC-OGC has no specific tumor markers or imaging characteristics

The increasing applications of serum tumor markers in PUC-OGC patients are helpful for screening patients; however, more specific diagnostic testing is needed. Among the four patients in this study, only one patient showed a slight increase in preoperative carcinoembryonic antigen, and one patient had a slight increase in preoperative CA19-9. The remaining tumor markers were negative. Preoperative abdominal ultrasonography and enhanced CT examination are helpful to make a definite diagnosis of malignancy. CT scan images can clearly show the size and location of the mass. Typical PUC-OGC can be observed by evident enhancement in the arterial phase, continuous enhancement of irregular solid and cystic solid masses in the portal vein phase (4), weak or low signal shadows, and unclear tumor boundaries. In this study, an abdominal enhanced CT scan in one patient revealed pancreatic morphological changes accompanied by space occupation in the tail of the pancreas which showed uneven signal shadows (Suppleemnatry Figure S4). MRI images showed that the tumor exhibited heterogeneous low-signal shadows on T1MI and T2MI. Part of the area around the tumor showed dark signals, but the center of the tumor showed slightly higher signals on T2MI. After injection of contrast agent, the tumor showed low levels of enhanced signal shadow in the arterial phase, portal vein phase and delayed phase (5). MRI can provide better anatomical detail than non-enhanced CT commonly used in PET-CT (6). CT- or EUS-guided fine needle aspiration (FNA) is also effective and accurate method for tumor diagnosis. However, preoperative FNA can increase the incidence of postoperative complications, and accurate preoperative diagnosis should not be blindly pursued without regard for the long-term complications, therefore appropriate cases should be selected.

### Characteristics of PUC-OGC gene mutation sites

Waddell et al. (7) classified PDAC into four subtypes based on potential clinical utility according to exome and copy number variation (CNV) analyses, including stable, locally rearranged, scattered and unstable. In the stable subtype, tumor genomes showed evidence of ≤50 structural variations that was located randomly throughout the genome. The locally rearranged type exhibited at least 50 focal variations on one or two chromosomes, and nearly 1/3 of the tumors of this subtype contained regions of copy number gain that harbored certain oncogenes. The scattered subtype exhibited non-random chromosomal damage and fewer than 200 structural variations. The unstable subtype exhibited a large number of structural variations (>200), and the high level of genomic instability suggested defects in DNA maintenance and potential, which may serve as sensitivity to DNA-damaging agents. Furthermore, researchers have identified five new susceptibility loci for pancreatic cancer in the Chinese population to provide effective markers for the early screening and diagnosis of this very malignant cancer (8). In this case, WES analysis revealed that the CNV in the KRAS gene had approximately 11.74% variation. Based on the mutational landscape of the pancreatic cancer cases described above, the case in this study should be classified as the stable subtype owing to the presence of less than 50 structural variation events in the CNV.

### KRAS mutation

According to TCGA database (cBioPortal), the KRAS gene has a mutation rate of 86%–95% in pancreatic cancer. It was reported that KRAS gene mutations in pancreatic cancer are found in more than 85% of all cases (9). Genetic and biochemical studies have shown that the KRAS-mediated RAS signaling pathway plays a key role in disease initiation, progression, and drug resistance. RAS signaling affects several cellular processes in PDAC, including cell proliferation, migration, cell metabolism, and autophagy. Ninety percent of pancreatic cancer patients have somatic oncogenic point mutations in KRAS that lead to its constitutive activation of the molecule. Although the activation level of each signal arm is different across different tumors and may be different even in different subclones of a single tumor, RAS activated Raf/MEK/ERK, PI3K/Akt/mTOR and Rala/B signaling pathways are active in human pancreatic cancer, cancer cell lines and mouse PDAC models. Because there are many kinds of KRAS mutations at the gene level, either during posttranslational maturation or after interaction with nucleotides and activation of various carcinogenic signals, the existence of KRAS mutations may also have therapeutic significance. Recently, some targeted therapies for MEK, ERK, PI3K and mTOR have been tested in mouse models of pancreatic cancer cell lines and diseases. The results show that these treatments can inhibit cell growth or delay tumor formation, and some inhibitors are currently in clinical trials, which is expected to bring new opportunities for the treatment of such patients (10). At this stage, the problem of small synthetic molecules remains as follows; their bioavailability allows for oral administration, but their toxicity and pharmacodynamics are unique. Thorough pharmacokinetic and toxicological studies are needed before they can be added to the treatment of PDAC.

### TP53 mutation

The TP53 gene encodes the tumor suppressor protein p53, which contains the N-terminal transactivation domain, the DNA-binding core domain, the tetramer domain and the C-terminal regulatory domain. The p53 protein inhibits the formation of urea by downregulating the key enzymes CPS1, OTC and ARG1 of the urea cycle, leading to the accumulation of ammonia, which affects the synthesis of polyamines and thus inhibits the proliferation and growth of tumor cells (11). According to the TCGA database (cBioPortal), mutations in the TP53 gene are 33%–70% of all found in pancreatic cancer. A study of 57 patients with pancreatic ductal adenocarcinoma evaluated TP53 mutations and mRNA expression (12), and the results show that patients with low TP53 mRNA expression had a poor prognosis (*p* = 0.032), which was more significant in individuals carrying the wild-type p53 gene (*p* = 0.021). According to the genetic test results of the patient in case 4 we reported, KRAS and TP53 mutations are the main driving forces leading to downstream signaling pathway activation and highly malignant disease. In addition, it shows that KRAS and TP53 mutations in PUC-OGC and PDAC can lead to the activation of oncogenes, thus targeted drugs for KRAS and TP53 oncogenic mutations are urgently needed.

### TMB

Tumor mutation burden (TMB) is generally defined as the total number of somatic non-synonymous mutations per million bases in the genome of each tumor patient. These new mutations produce tumor-specific antigens and are an important factor in activating tumor-specific T cell responses (13). The dismatch repair (MMR) system plays an important role in the repair of DNA sequence mismatches during replication. Defects in the MMR system can cause DNA replication errors, which can lead to a high TMB or an increase in MSI elevations (14). Genomic analysis with a large sample size of 3594 cases of PADC (15) confirmed that only 0.5% of the samples had a high MSI/high TMB status. Salem et al. analyzed 870 cases of PDAC and found that the prevalence of high TMB in PDAC was low (1.4%) and in most cases had high/low MSI (16). PDAC also has an immunosuppressive tumor microenvironment with highly programmed cell death ligand 1 (PD-L1) expression, which in turn inhibits the cytotoxicity of activated T cells (17). Several studies have shown that PDAC patients with high PD-L1 expression have a significantly poorer prognosis than in all PDAC patients compared to those without PD-L1 expression (17–22). In Luchini’s study, PD-L1 was expressed in tumor cells in 17 of 27 PUC-OGC cases (63%) and was more common in patients with associated PDAC (*p* = 0.04). The expression of PD-L1 was associated with a poor prognosis. Multivariate analysis confirmed that the all-cause mortality rate of PD-L1-positive PUC-OGC was three times that of PD-L1-negative PUC-OGC (hazard ratio, 3.397; 95% confidence interval, 1.023–18.375; *p* = 0.034) (23). It has been speculated that the possible mechanism of PD-L1-positive PUC-OGC aggression is that PD-L1 inhibits antitumor immunity and enables tumor cells to escape the cytotoxic activity of host T lymphocytes. This hypothesis is related to the fact that PUC-OGC is rich in inflammatory cells within the tumor components. This is also consistent with the results of Lawrie K et al., who found that PUC-OGC has significantly more CD3^+^ and CD8^+^ TILs/mm^2^ than traditional PDAC (24). Clinical studies have shown that TMB can be used as the latest marker to evaluate the efficacy of immunotherapy. The immune system is more likely to recognize tumor cells with a higher level of TMB and which could trigger a stronger immune response to checkpoint inhibitors. However, the tumor mutation load of patient 4 in this study was low and they did not show a high MSI, and the expression of PD-L1 was negative, therefore the patients included in this study did not have indications for immunotherapy.

### Therapy

Given the low disease incidence and paucity of available clinical outcome data, evidence-based guidelines for the treatment of PUC-OGC do not exist, even as part of broader PDAC treatment guidelines. In the United States, neither the NCCN Clinical Practice Guidelines in Oncology (NCCN Guidelines) for PDAC nor the ASCO guidelines for curable or metastatic PDAC offer guidance on PUC-OGC treatment (25). However, the role of adjuvant therapy is still unclear. Given the radiosensitivity of giant cell tumors of the bone (26), abdominal radiation therapy for pancreatic giant cell cancer is also theoretically beneficial, which is the basis for the decision to proceed with radiation therapy. Yoshioka M et al. reported a case of undifferentiated pancreatic cancer diagnosed as osteoclast-like giant cells accompanied by massive portal vein tumor thrombosis (PVTT). The patient first underwent distal pancreatectomy and tumor thrombectomy to prevent life-threatening portal vein obstruction. The patient has achieved in complete remission for 12 months and had survived at least 19 months since the surgery (27). However, Robert J. Besaw was the first to demonstrate the continued response of pancreatic PUC-OGC to PD-1/PD-L1 blockade even without resection (28).

### Prognosis

**FIGURE 5:**
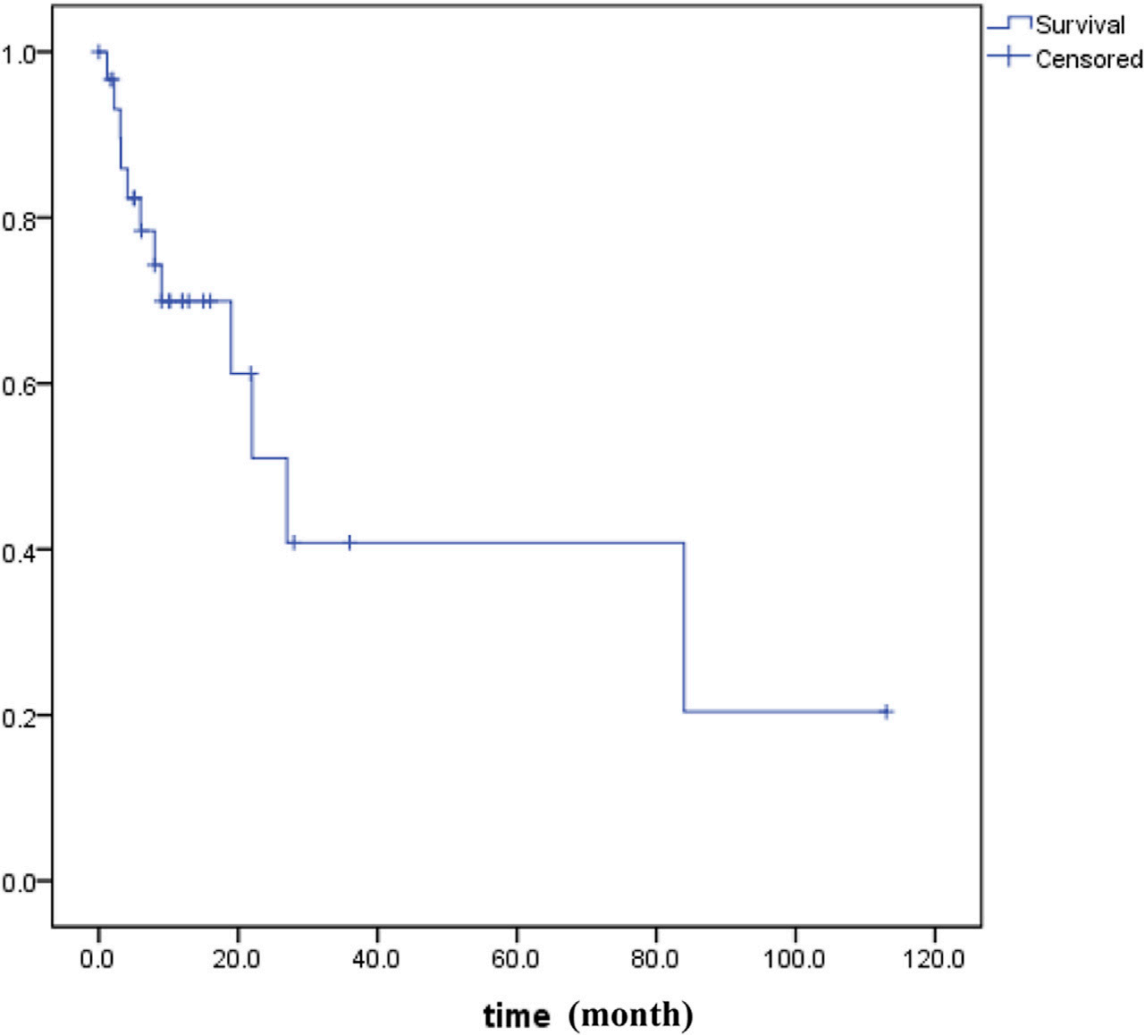
Kaplan–Meier survival data for patients (*n* = 31) with OGCT of the pancreas.

PUC-OGC is relatively rare in clinical practice. It has been reported that PUC-OGC has a significantly different prognosis, with a median survival time of 11 months, while that for non-resectable cases decreases to 6.5 months (29,30). We searched all literature in the PubMed, WOS, EMBASE and CNKI before October 2020 and performed all patients’ survival analyses for all of PUC-OGC patients (Figure 5). A total of 31 eligible patients were included, comprising 16 male patients and 15 female patients. The median age of diagnosis was 62 years old, and the median survival time was 11 months, with an average survival time of approximately 24.45 months.

According to our study, the median survival time of the 4 patients was 9 months, and all cases of them were resectable. It has been reported that the prognosis of resectable PUC-OGC is better than that of undifferentiated carcinoma without OGC, which may be due to the slow rate of metastasis and minimal metastasis to the lymph nodes. The degree of malignancy and prognosis of the tumor are not related to the number of giant cells. The factors that affect prognosis are still being investigated. If the tumor does not invade other organs and has no lymph node metastasis, the selection of an appropriate surgical method and individualized and precise adjuvant treatment after operation may prolong the survival time to a certain extent.

## Conclusion and prospects

Early diagnosis of PUC-OGC is difficult because of its occult onset, and the lack of specific clinical manifestations and blood tumor markers. Pathological analysis is essential to the diagnosis and treatment of cancer. Knowledge of the different histological types of pancreatic carcinoma and the targeted treatment after a clear diagnosis can contribute to prognosis of pancreatic carcinoma patients. In this article, we not only summarize the pathological and genetic characteristics of PUC-OGC but also provide a deeper understanding of pancreatic cancer for future reference. With the development of molecular biology and the continuous advancement in gene detection technology, it may be possible to uncover the genetic characteristics of PUC-OGC in the future, uncover its mystery, and create a foundation to bring new opportunities to manage for PUC-OGC *via* molecular diagnosis, surgery, chemoradiotherapy, targeted therapy, and immunotherapy among other aspects.

## Data Availability

The data analyzed in this study is subject to the following licenses/restrictions: He datasets used and/or analyzed during the current study are available from the corresponding author on reasonable request. Requests to access these datasets should be directed to 17839223815@163.com.

## Abbreviations

AFP, blood alpha-fetoprotein; AR, androgen receptor; CA, cytokeratin; FNA, fine needle aspiration; CA-125, glycemic-like antigen 125; CA19-9, glycemic antigen 19-9; CEA, blood carcinoma embryo antigen; CK, cytokeratin; CT, Computed Tomography; DES, desmin; dMMR, dismatch repair; EMA, epithelial membrane antigen; FNA, fine needle aspiration; NGS, Next-Generation Sequencing; PDAC, pancreatic ductal adenocarcinoma; PD-L1, programmed cell death ligand 1; PUC-OGC, Pancreatic undifferentiated carcinoma with osteoclast-like giant cells; PVTT, portal vein tumor thrombosis; SMA, alpha-smooth muscle actin; TMB, Tumor mutation burden; Vim, Vimentin.
