# Supplementary material for: Case report: Pathological and genetic features of pancreatic undifferentiated carcinoma with osteoclast-like giant cells
Source: Pathol Oncol Res. 2023 Mar 3;29:1610983. doi: 10.3389/pore.2023.1610983 (PMC10021297; doi:10.3389/pore.2023.1610983)
Supplement: Supplementary file 1 [file Table1.DOCX]

| Summary of test results | | |
| --- | --- | --- |
| Testing significance | Testing items | Testing results |
| Targeted therapy, prognosis and drug resistance related | Somatic cell mutation | AR、FBXW7、CCNE1、BTK、KRAS、T53 |
|  | Germ cell mutation | None |
| PARP inhibitors | BRCA1/2 mutation | None |
|  | HRD score | None |
| DNA damage repair pathway | The gene of DNA damage repair pathway | None |
| Immunotherapy | PD-L1 test result | negative（TP53<1%，CPS=5） |
|  | Mutation | 3.35Muts/Mb low （lower than 76% of pancreatic patients） |
|  | MSI | MSS |
|  | EBV | Negative |
|  | HLA | Heterogeneous |
|  | Positive correlation genes | KRAS、TP53 |
|  | Negative correlation genes | None |
