# Supplementary figures and images for: Case report: Pathological and genetic features of pancreatic undifferentiated carcinoma with osteoclast-like giant cells
Source: Pathol Oncol Res. 2023 Mar 3;29:1610983. doi: 10.3389/pore.2023.1610983 (PMC10021297; doi:10.3389/pore.2023.1610983)

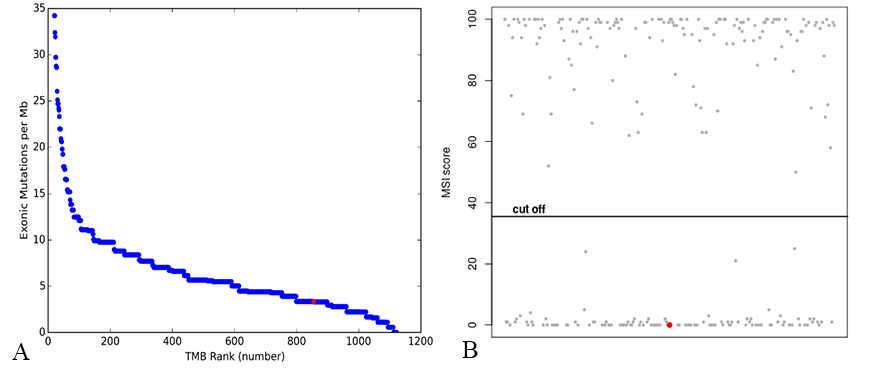

Supplement: Supplementary file 2 [file Image3.TIF]

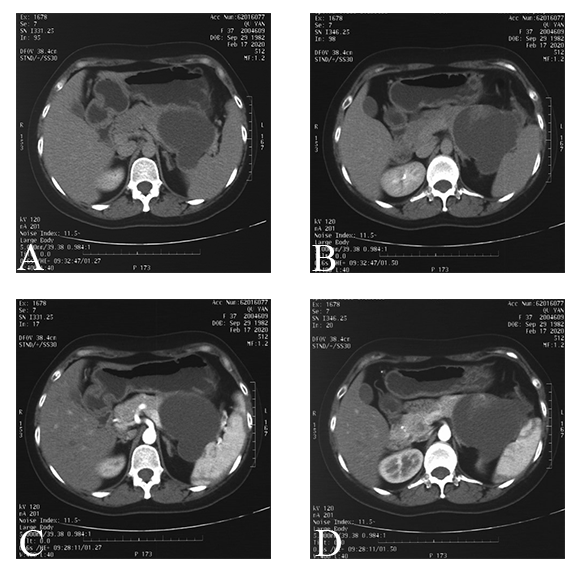

Supplement: Supplementary file 3 [file Image4.TIF]

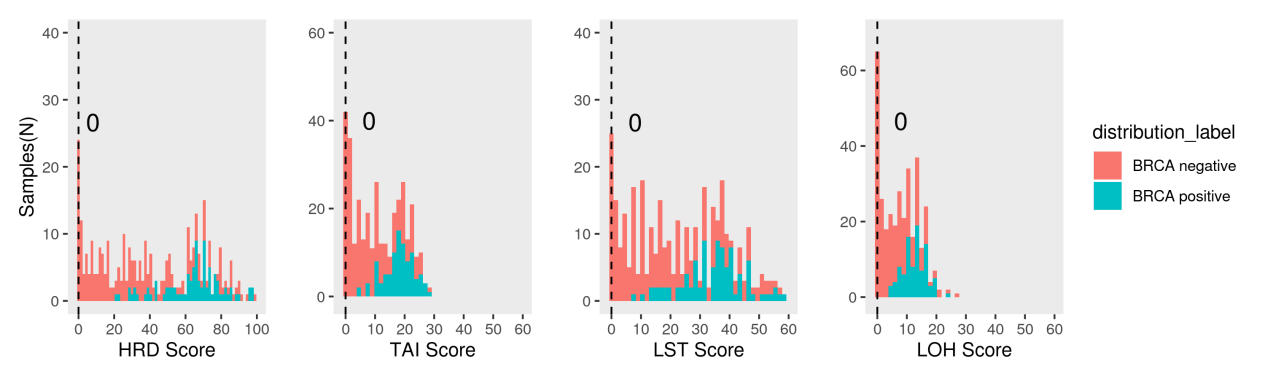

Supplement: Supplementary file 4 [file Image2.TIF]

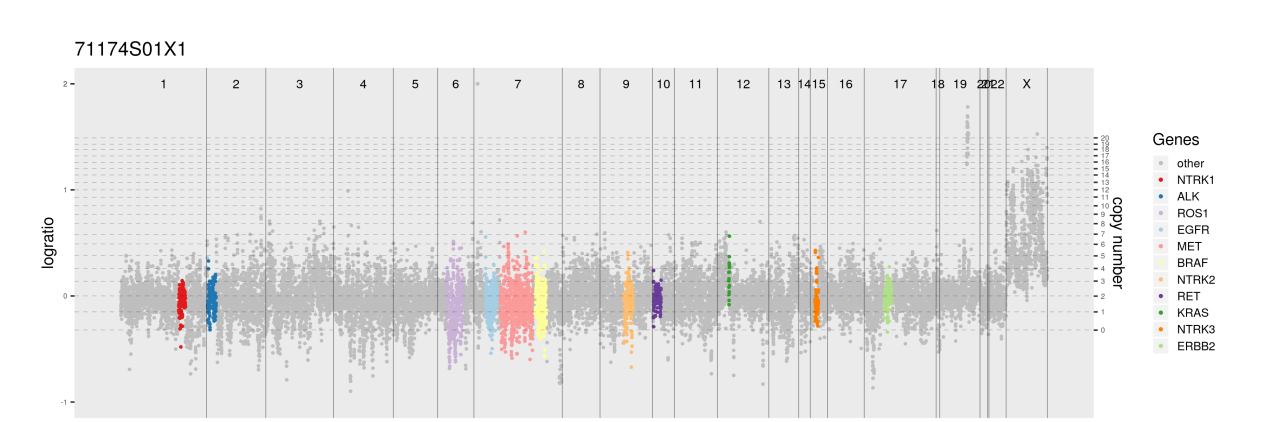

Supplement: Supplementary file 5 [file Image1.TIF]
