# Supplementary material for: Case report: Pathological and genetic features of pancreatic undifferentiated carcinoma with osteoclast-like giant cells
Source: Pathol Oncol Res. 2023 Mar 3;29:1610983. doi: 10.3389/pore.2023.1610983 (PMC10021297; doi:10.3389/pore.2023.1610983)
Supplement: Supplementary file 6 [file Table2.DOCX]

| Somatic cell mutation | | |
| --- | --- | --- |
| Gene | results | Mutation abundance/copy number |
| AR | Copy number increase | 9 |
| FBXW7 | p.R465H Exon9 | 4.09% |
| CCNE1 | Copy number increase | >20 |
| BTK | Copy number increase | 10 |
| KRAS | p.G12R Exon2 | 11.74% |
| TP53 | p.P75Lfs*48 Exon5 | 6.10% |
